# Dose-dependent effects of testosterone on proteins related to nitric oxide signaling pathway and trophic factors in the spinal cord of adolescent trained rats

**DOI:** 10.3389/fspor.2025.1635517

**Published:** 2025-07-22

**Authors:** Katarzyna Nierwińska, Konstancja Grabowska, Małgorzata Chalimoniuk, Sławomir Jagsz, Józef Langfort, Andrzej Małecki, Marta Nowacka-Chmielewska

**Affiliations:** ^1^ Laboratory of Molecular Biology, Institute of Physiotherapy and Health Sciences, The Jerzy Kukuczka Academy of Physical Education in Katowice, Katowice, Poland; ^2^ Department of Basic Biomedical Sciences, Institute of Sport Sciences, The Jerzy Kukuczka Academy of Physical Education in Katowice, Katowice, Poland; ^3^ Department of Physical Education and Health in Biala Podlaska, Faculty in Biala Podlaska, Jozef Pilsudski University of Physical Education in Warsaw, Biala Podlaska, Poland; ^4^ Department of Sports Nutrition, The Jerzy Kukuczka Academy of Physical Education in Katowice, Katowice, Poland

**Keywords:** endurance training, testosterone, neurotrophins, NO, NOS, adolescent rats, spinal cord

## Abstract

**Introduction:**

Endurance training plays an important role in, for example, triathlon, marathon, or road cycling and in combination with strength training. Adolescence has been associated with increased interest among of young people, especially boys, in strength-related and endurance sports or body-building. Anabolic androgen steroid use is a public health threat. The present study aimed to estimate the effect of endurance training, two doses of testosterone, and the combination of these stimuli on the level and activity of proteins related to the nitric oxide (NO) signaling pathways in the spinal cord in adolescent male rats.

**Methods:**

Adult male Wistar rats were trained using a motor-driven treadmill for 6 weeks (40–60 min, 5 times per week) and/or were treated for 6 weeks with two doses of testosterone (i.m.; 8 mg/kg or 80 mg/kg body weight). At the end of the experiment, spinal cord samples were collected for further evaluation.

**Results and Discussion:**

Major findings from the study are that a high dose of testosterone increases proteins related to the NO signaling pathway (eNOS, nNOS, CGβ1, PKC), but decreases trophic factors (BDNF, VEGF) and p-Akt. Endurance training by itself increases the spinal protein levels of CGβ1, VEGF, and kinases -p-Akt and PKC, but decreases kinase p-p38 MAPK; and the combination of endurance training and high doses of testosterone enhances changes in the protein level of nNOS, p-p38 and p-Akt. In conclusion, at least some of the effects of endurance training and testosterone may be related to the intensity of NO-related signal transmission and protein kinase systems.

## 1 Introduction

Endurance training characterized by enhanced cardiac output, maximal oxygen consumption, and mitochondrial biogenesis has been reported to improve both central and peripheral tissues allowing for enhanced exercise economy and a greater ability for an individual to run for longer distances and times (1, 2). Most activities combine endurance and strength; this type of training has been termed concurrent exercise (2). Therefore, endurance training plays an important role in endurance sports (for example triathlon, marathon, or road cycling) and in combination with strength training (3). Adolescence has been associated with increased interest of young people, especially boys, in strength-related and endurance sports or body-building (4, 5). Results from a cross-national European study by Kokkevi et al. (2008) showed that daily exercising appears to increase the risk of anabolic steroid use in adolescents (6). Simultaneously, anabolic androgen steroid users had more anger issues, anxiety, and depression, and their self-esteem was lower than that of non-anabolic androgen steroid users (7). In light of the studies mentioned above, anabolic androgen steroid use is a public health threat. The effects of many other potential risk factors have not been fully clarified.

In the present study, we tested the effects of this combination (endurance training and testosterone supplementation) on proteins related to the nitric oxide (NO) signaling pathways in the spinal cord in adolescent male rats. In vertebrates, neural mechanisms at different levels of the CNS participate in the control of locomotion. Proper locomotion requires effective coordination of the limbs which is achieved through a complex interplay between spinal circuits and supraspinal structures (8, 9). Neuronal networks of central pattern generators located in the spinal cord underlie the production of various rhythmic patterned outputs that drive rhythmic locomotor movements (e.g., swimming, cycling, or running) (10, 11). At the molecular level, locomotor movements may be modulated by NO, a highly conserved signaling molecule that affects many CNS signaling processes and involves neurotransmitters and neuromodulators (12, 13). Enzyme synthesizing NO termed nitric oxide synthase (NOS) has many isoforms, which differ significantly in tissue distribution, and expression patterns and are encoded by different genes widely distributed (13). Three isoforms of NOS are important for NO production in the CNS: neuronal (nNOS), endothelial (eNOS), and inducible (iNOS). Whereas expression of the latter is associated with (pro)inflammatory states, it was shown that nNOS and eNOS are expressed constitutively (14, 15). All three isoforms of NOS have several phosphorylation sites for different protein kinases, including PKA (Protein kinase A), PKC (Protein kinase C), AKT (known as Protein kinase B), and CAMK (Ca2+–calmodulin-dependent kinase) (16). The best-characterized NO target in neurons is soluble guanylyl cyclase (sGC), which after activation increased 3',5'-cyclic guanosine monophosphate levels to activate cGMP-dependent protein kinases (PKGs) (13). Both eNOS and nNOS can be significantly upregulated by physical exercise/aerobic training (17, 18). It was shown that NO might play a role in enhanced synthesis of the brain BDNF (brain-derived neurotrophic factor) in response to exercise (19). NO, and BDNF can also mutually influence their production and synaptic availability in cultured rodent neocortical neurons (20). Moreover, elevated expression of the hippocampal BDNF in rodents was proposed as one of the key mechanisms underlying exercise-induced brain plasticity and cognitive enhancement (21). Finally, improved learning and memory in the exercised adolescent rats were associated with changes in the brain levels of BDNF and VEGF (vascular endothelial growth factor) (22). Hence, we compared the protein levels of various NOS isoforms, the expression of the β1 subunit of CG (CGβ1), different protein kinases, and BDNF and VEGF in the spinal cord of sedentary and endurance-trained young male rats administered testosterone for 6 weeks. The spinal cord was chosen due to its important role in transferring information between the body and the brain, as well as a crucial region involved in the integration and coordination of locomotion.

## 2 Material and methods

### 2.1 Animals and experimental groups

The animals utilized in this study were sourced from the M. Mossakowski Institute of Experimental and Clinical Medicine at the Polish Academy of Sciences in Warsaw, Poland. Their handling adhered to the guidelines outlined in Directive 2010/63/EU concerning animal experimentation. All protocols were reviewed, approved, and supervised by the Local Ethics Committee for Animal Experimentation in Warsaw (protocol number 64/2009). The study utilized the minimum number of rats necessary to ensure reliable results, and every possible measure was taken to reduce the animals’ distress. The research involved 6-week-old male Wistar rats with a body weight ranging between 100 and 120 grams. These rats were kept in groups of 3–4 per cage within a temperature-controlled environment (maintained at 20–22°C and relative humidity of 55 ± 10%). The light-dark cycle in the housing facility was set at 12 h each, starting at 7:00 a.m., and the rats had unrestricted access to food and water. The study followed a previously described protocol (23, 24), which involved a combination of endurance training and administration of testosterone propionate (TP) over six weeks. Before commencing the main experiment, all rats underwent a habituation period on a motorized treadmill for rodents. Over three consecutive days, they were tested for their ability to run (sessions of 3, 5, and 9 min with 15-minute breaks in between). Rats that refused to participate in the running sessions were excluded from the study. Due to the specific administration protocols for testosterone and exercise interventions, blinding during the treatment phase and outcome collection was not feasible; however, data analysis was performed by an investigator blinded to group allocation to minimize bias. The remaining rats were then randomly assigned to various research groups:

• Sed: vehicle (*n* = 20): sedentary (untrained) animals, receiving sesame oil (vehicle) intramuscularly (im.);
• Sed: T [8 mg/kg] (*n* = 19): sedentary rats receiving TP im. TP at a low dose of 8 mg/kg;
• Sed: T [80 mg/kg] (*n* = 20): sedentary rats receiving im. TP at a high dose of 80 mg/kg;
• EndTr: vehicle (*n* = 20): trained rats receiving sesame oil (vehicle) im.;
• EndTr: T [8 mg/kg] (*n* = 19): trained rats receiving im. TP at a low dose of 8 mg/kg;
• EndTr: T [80 mg/kg] (*n* = 20): trained rats receiving im. TP at a high dose of 80 mg/kg.

To minimize potential confounders, all animals were age- and strain-matched, housed under identical environmental conditions, and subjected to standardized feeding and handling protocols throughout the experimental period.

### 2.2 Testosterone administration

A stock solution of testosterone propionate (TP) obtained from Jelfa, Poland was diluted with sesame oil as required before intramuscular injections. The injections were administered weekly over a six-week period, alternating between the left and right hind limbs. The TP dosage was determined based on the methodology described by Sadowska et al. (23). To ensure consistency, the TP solution in sesame oil was prepared fresh before each injection. For control groups, an equivalent volume of sesame oil was administered on the same schedule.

### 2.3 Endurance training

The rats assigned to endurance training underwent exercise sessions on a flat-surfaced rodent treadmill five days a week for six weeks. The initial treadmill speed was set at 16 m/min for the first week and subsequently increased by 4 m/min each week over the next three weeks, stabilizing at 28 m/min for the remainder of the training period. The daily training duration started at 40 min per session during the first week and was extended by 5 min per day over the following four weeks, ultimately reaching 1 h per day for the last two weeks. No form of negative reinforcement, such as electric shocks, was utilized during the training.

### 2.4 Tissue collection

Two days after completing the training program, the rats were sacrificed by decapitation, and their spinal cords were extracted. The extracted spinal cords were manually homogenized at 4°C using a glass Dounce homogenizer with thirteen piston strokes. The isolation buffer contained a combination of 1 M TRIS-HCl, 5 M NaCl, 10% SDS, 0.2 M EDTA, Igepal, PMSF (10 mg/ml), aprotinin (5 mg/ml), pepstatin (2 mg/ml), leupeptin (1 mg/ml), 0.1 M Na3VO4, and bidistilled water. The homogenized lysate was centrifuged at 4°C for 60 min at 15,000 × g. The supernatant (cytosolic fraction) obtained from centrifugation was processed using the western blot technique for subsequent analysis. After determining the protein concentration with the Bradford method, the samples were stored at −80°C.

### 2.5 Determination of nitrogen oxide synthase (NOS) activity

The activity of NOS was assessed by quantifying the radioactive [14C] L-citrulline produced in equimolar amounts to NO from labeled [14C]L-arginine in a NOS-catalyzed reaction in the presence of necessary cofactors. A total of 500 µg of homogenate was combined with a reaction mixture containing 50 mM Tris-HCl buffer (pH 7.4), 100 µM [U-14C]L-Arginine (0.2 µCi), 2 mM CaCl2, 1 µM calmodulin, 15 µM FAD, 10 µM tetrahydrobiopterin, 1 mM NADPH, 1 mM EDTA, and 1 mM DTT, in a final volume of 300 µl. The mixture was incubated for 30 min at 37°C. To terminate the reaction, 1 ml of chilled 100 mM Tris-HCl buffer containing 10 mM EGTA (pH 5.5) was added. Following 5 min of incubation at 4°C, the mixture was centrifuged at 300 g for 10 min, and the supernatant was applied to a Dowex AG 50W-X8 column (Na + form). The eluate, collected in 1 ml aliquots with bidistilled water, was analyzed for radioactivity in 0.5 ml samples.

### 2.6 Western blot

For protein analysis, 50 µg of total protein was separated on 8% SDS-polyacrylamide gels and subsequently transferred onto nitrocellulose membranes (Bio-Rad Laboratories, Inc.) using a current of 250 mA for 1.5 h at 4°C. To block nonspecific binding, the membranes were incubated overnight at 4°C with 5% fat-free milk in a Tris-buffered solution. Monoclonal antibodies, procured from Santa Cruz Biotechnology, USA, served as primary antibodies, as outlined in Table 1. The membranes were washed and treated with a secondary goat anti-rabbit IgG antibody (1:5,000; Abcam, ab97051). The immunoblots were visualized using ECL reagents (Amersham Pharmacia Biotech, Inc.) and detected using a western blot detection system (Amersham Pharmacia Biotech, Inc.). Orginal full-length and blots were included in the Additional file 1. The resulting bands were quantified using the ImageJ software (NIH, Bethesda, MD, USA). Results were normalized against the loading control (β-actin, 1:1,000, Santa Cruz Biotechnology) and expressed as the mean ± SD of fold changes relative to the control group (% of control values).

**Table 1:** Summary of primary antibodies.

Primary antibody	Final dilution
Anti-BDNF	1:200 (dilution)
Anti-CGβ1	1:200 (dilution)
Anti-eNOS	1:200 (dilution)
Anti-nNOS	1:200 (dilution)
Anti-p-Akt	1:100 (dilution)
Anti-PKC	1:100 (dilution)
Anti-p-p38	1:100 (dilution)
Anti-VEGF-A	1:100 (dilution)
Anti-VEGF-C	1:100 (dilution)

### 2.7 Statistical analysis

Prism 10.4.0 (GraphPad Software, San Diego, CA, USA) was used for statistical analyses and figure generation. The results are expressed as the means and standard deviation (SD). The distribution of each dataset was checked for normality using the Shapiro–Wilk test. Western blot results were analyzed using a two-way analysis of variance (ANOVA) for the following factors: endurance training [sedentary (untrained) vs. trained] and treatment [vehicle vs. T (8 mg/kg) and vehicle vs. T (80 mg/kg)], followed by Tukey's multiple comparisons tests when appropriate. Differences were considered to be statistically significant when *p* < 0.05.

Statistical details regarding western blot analysis are summarised in Table 2.

**Table 2:** Statistical source data for extended data from the ANOVA test.

Protein	Two-way ANOVA		
	F [DFn, DFd], *p*-value for testosterone treatment	F [DFn, DFd], *p*-value for training	F [DFn, DFd], *p*-value for testosterone x training
BDNF	F[2,12] = 19.97, *p* = 0.0002	F[1,12] = 1.706, *p* = 0.216	F[2,12] = 0.352, *p* = 0.709
CGβ1	F[2,12] = 218.3, *p* < 0.0001	F[1,12] = 34.91, *p* < 0.0001	F[2,12] = 19.80, *p* < 0.0001
eNOS	F[2,12] = 19.76, *p* = 0.0002	F[1,12] = 1.088, *p* = 0.317	F[2,12] = 1.355, *p* = 0.294
nNOS	F[2,12] = 26.01, *p* < 0.0001	F[1,12] 6.341, *p* = 0.0270	F[2,12] = 7.752, *p* = 0.0069
p-Akt	F[2,12] = 430.5, *p* < 0.0001	F[1,12] = 164.9, *p* < 0.0001	F[2,12] = 91.56, *p* < 0.0001
PKC	F[2,12] = 14.39, *p* = 0.0007	F[1,12] = 0.818, *p* = 0.383	F[2,12] = 12.54, *p* = 0.0011
p-p38	F[2,12] = 25.34, *p* < 0.0001	F[1,12] = 46.35, *p* < 0.0001	F[2,12] = 36.05, *p* < 0.0001
VEGF-A	F[2,12]= 63.09, *p* < 0.0001	F[1,12] = 21.78, *p* = 0.0005	F[2,12] = 3.803, *p* = 0.0526
VEGF-C	F[2,12] = 4.525, *p* = 0.0343	F[1,12] = 95.36, *p* < 0.0001	F[2,12] = 5.874, *p* = 0.0166

## 3 Results

### 3.1 Testosterone in both doses and training alone increased NOS activity but a combination of a high dose of testosterone with training induced an opposite effect

**Figure 1:**
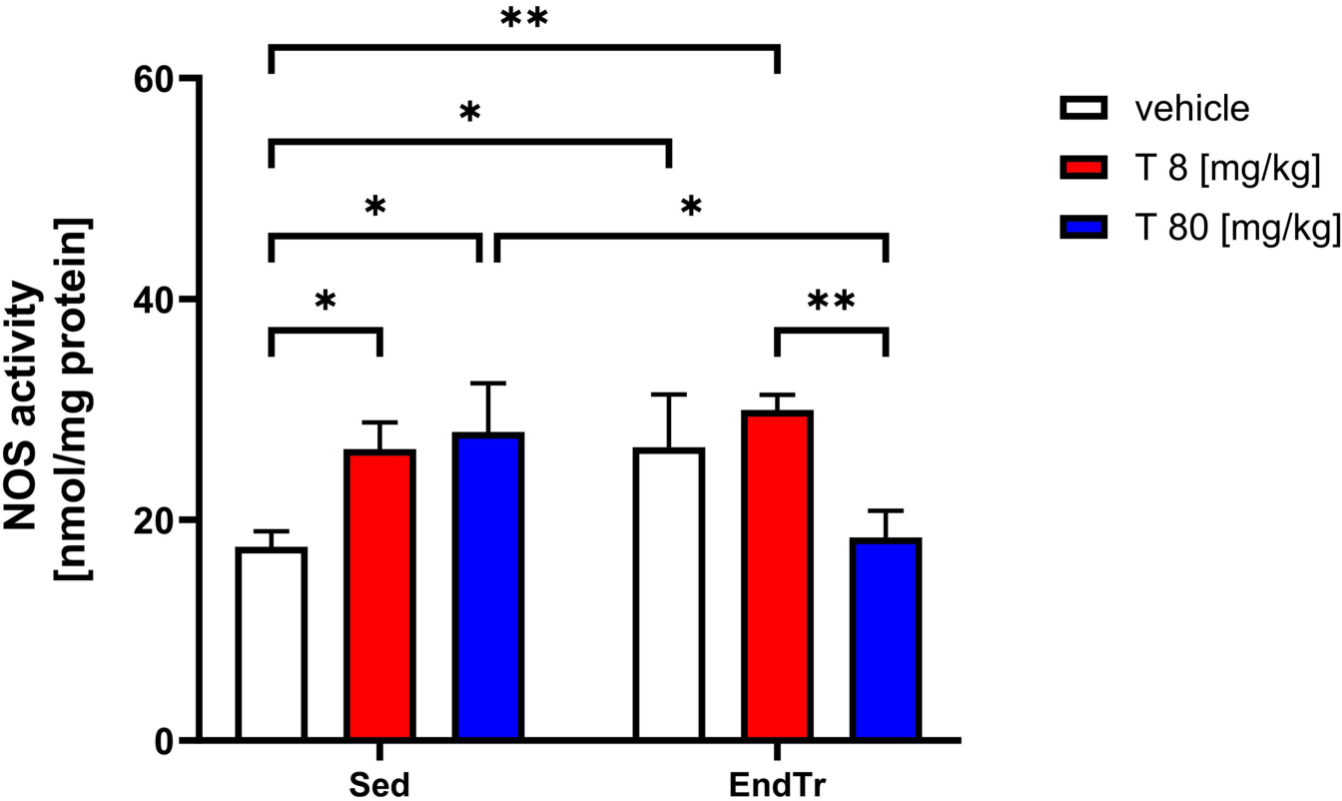
Six weeks of endurance training and testosterone treatment alter NOS activity in the spinal cord of male adolescent rats. Data were analyzed with a two-way ANOVA and *post-hoc* Tukey's test: **p < 0.01, *p < 0.05 (*n* = 19-20 for each experimental group).

The two-way ANOVA revealed significant effects of endurance training [F(1,12) = 21.54, *p* = 0.0001], and testosterone treatment on NOS activity (F[1,12] = 8.091, *p* = 0.0148), but no interaction between these factors (F[2,12] = 0.833, *p* = 0.458, Figure 1, Table 2). NOS activity in the spinal cord was significantly increased by both low and high doses of testosterone, by endurance training alone, and by the combination of training with a low testosterone dose, when compared to sedentary controls (Sed: veh vs. Sed: T 8 [mg/kg]: *p* = 0.0411; Sed: veh vs. Sed: T 80 [mg/kg]: *p* = 0.0147; Sed: veh vs. EndTr: veh: *p* = 0.0366; Sed: veh vs. EndTr: T 8 [mg/kg]: *p* = 0.0040). In contrast, combining training with a high dose of testosterone did not increase NOS activity. This group showed significantly lower NOS activity compared to sedentary rats treated with a high dose of testosterone [Sed: T 80 [mg/kg] vs. EndTr: T 80 [mg/kg]: *p* = 0.0026] and to trained rats treated with a low testosterone dose [EndTr: T 8 [mg/kg] vs. EndTr: T 80 [mg/kg]: *p* = 0.0068].

### 3.2 A high dose of testosterone, but no training enhanced the spinal level of eNOS

**Figure 2:**
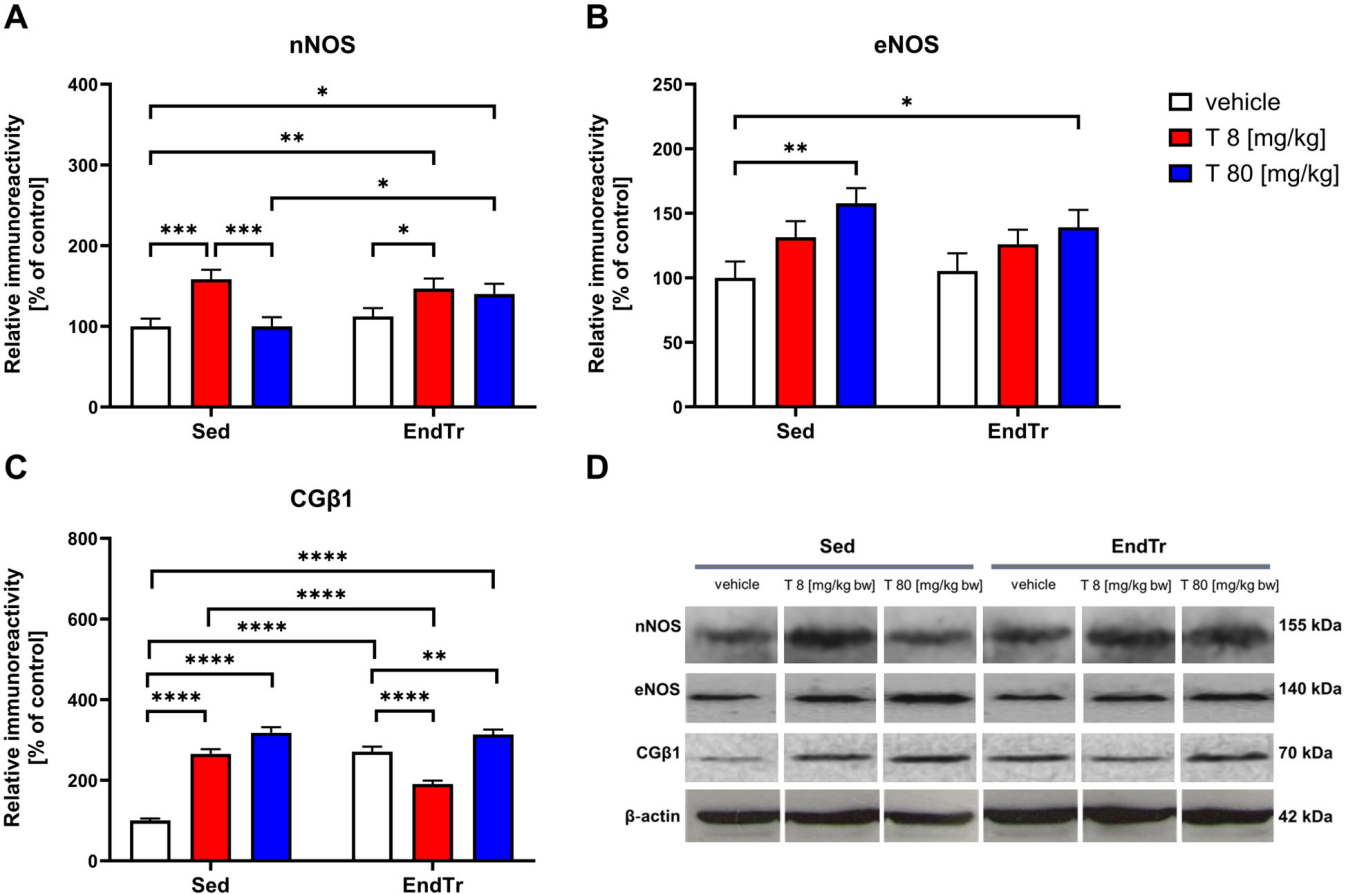
Six weeks of endurance training and testosterone treatment alter the spinal levels of proteins related to the NO pathway in male adolescent rats. Western blotting analysis of protein relative immunoreactivity of nNOS **(A)**, eNOS **(B)**, and CGβ1 **(C)** Testosterone affected nNOS levels in both sedentary and trained animals in a dose-dependent manner **(A)** A higher dose of testosterone (T 80 mg/kg) led to an increase in eNOS and CGβ1 levels (B, C). Testosterone treatment, either in low or high doses, as well as endurance training or the combination of both interventions, upregulated the CGβ1 level **(C)** Representative images of Western blotting analysis, cropped bands from different parts of the same blots are shown **(D)** Values are expressed as a percentage of the control. Data were analyzed with a two-way ANOVA and *post-hoc* Tukey's test: ****p < 0.0001, ***p < 0.001, **p < 0.01, *p < 0.05 (*n* = 19-20 for each experimental group).

We demonstrated significant effects of testosterone treatment on spinal levels of eNOS (F[2,12] = 19.76, *p* < 0.0002) with no changes in training effect (F[1,12] = 1.088, *p* = 0.317) or interaction of these factors (F[2,12] = 1.355, *p* = 0.294, Figure 2A, Table 2). High dose of testosterone increased the eNOS protein level of sedentary and trained rats compared to appropriate sedentary groups [Sed: veh vs. Sed: T 80 [mg/kg]: *p* = 0.0013, Sed: veh vs. EndTr: T 80 [mg/kg]: *p* = 0.0242].

### 3.3 Testosterone in both doses increased the nNOS protein level in trained rats

The two-way ANOVA revealed significant effects of endurance training (F[1,12] = 6.341, *p* = 0.027) and testosterone treatment on nNOS protein level (F[1,12] = 26.01, *p* < 0.0001), with a significant interaction between these factors (F[2,12] = 7.752, *p* = 0.0069, Figure 2B). We observed an increase in the nNOS protein level in rats receiving a low dose of testosterone, regardless of the training stimulus in comparison to vehicle-treated groups (Sed: veh vs. Sed: T 8 [mg/kg]: *p* = 0.0005; EndTr: veh vs. EndTr: T 8 [mg/kg]: *p* = 0.0291). An increase in the spinal nNOS levels was noted in combination with endurance training both with low and high doses of testosterone (Sed: veh vs. EndTr: T 8 [mg/kg]: *p* = 0.0033; Sed: veh vs. EndTr: T 80 [mg/kg]: *p* = 0.0106). Testosterone dose-dependent reductions in the level of nNOS protein were observed in the sedentary group (Sed: T 8 vs. Sed: T 80 mg/kg: *p* = 0.0005). We noted a significant difference in the nNOS protein level between training and non-training animals receiving a high dose of testosterone [Sed: T 80 vs. EndTr: T 80 (mg/kg): *p* = 0.01].

### 3.4 Testosterone in both doses and combination with training increased the CGβ1 protein level

Concerning CGβ1 protein levels, we demonstrated significant effects of endurance training (F[1,12] = 34.91, *p* < 0.0001), and testosterone treatment (F[2,12] = 218.3, *p* < 0.0001) and interaction between these factors (F[2,12] = 198.0, *p* < 0.0001, Figure 2C, Table 2). The *post hoc* analysis revealed that training increased CGβ1 protein levels in the vehicle-treated group (Sed: veh vs. EndTr: veh: *p* < 0.0001). A *post hoc* analysis showed that CGβ1 protein level increased either when sedentary animals were supplemented with low or high testosterone dose [*p* < 0.0001 for comparisons Sed: veh vs. Sed: T 8, and Sed: veh vs. Sed: T 80 (mg/kg)]. CGβ1 protein levels differed significantly in the low-dose testosterone groups depending on the training stimulus (Sed: T 8 [mg/kg] vs. EndTr: T 8 [mg/kg]: *p* < 0.0001). In trained animals, a significant decrease was observed in the low-dose treated group compared to the vehicle-treated group [EndTr: veh vs. EndTr: T 8 (mg/kg): *p* < 0.0001]. We noted that the spinal levels of CGβ1 increased when animals were supplemented with high testosterone dose, regardless of training stimulus in comparison to appropriate control groups [*p* < 0.0001 for comparisons Sed: veh vs. Sed: T 80, Sed: veh vs. EndTr: T 80, and EndTr: veh vs. EndTr: T 80 (mg/kg): *p* = 0.0046].

### 3.5 The opposite direction of changes induced by a low and high dose of testosterone alone and in combination with training on the p-Akt protein level

**Figure 3:**
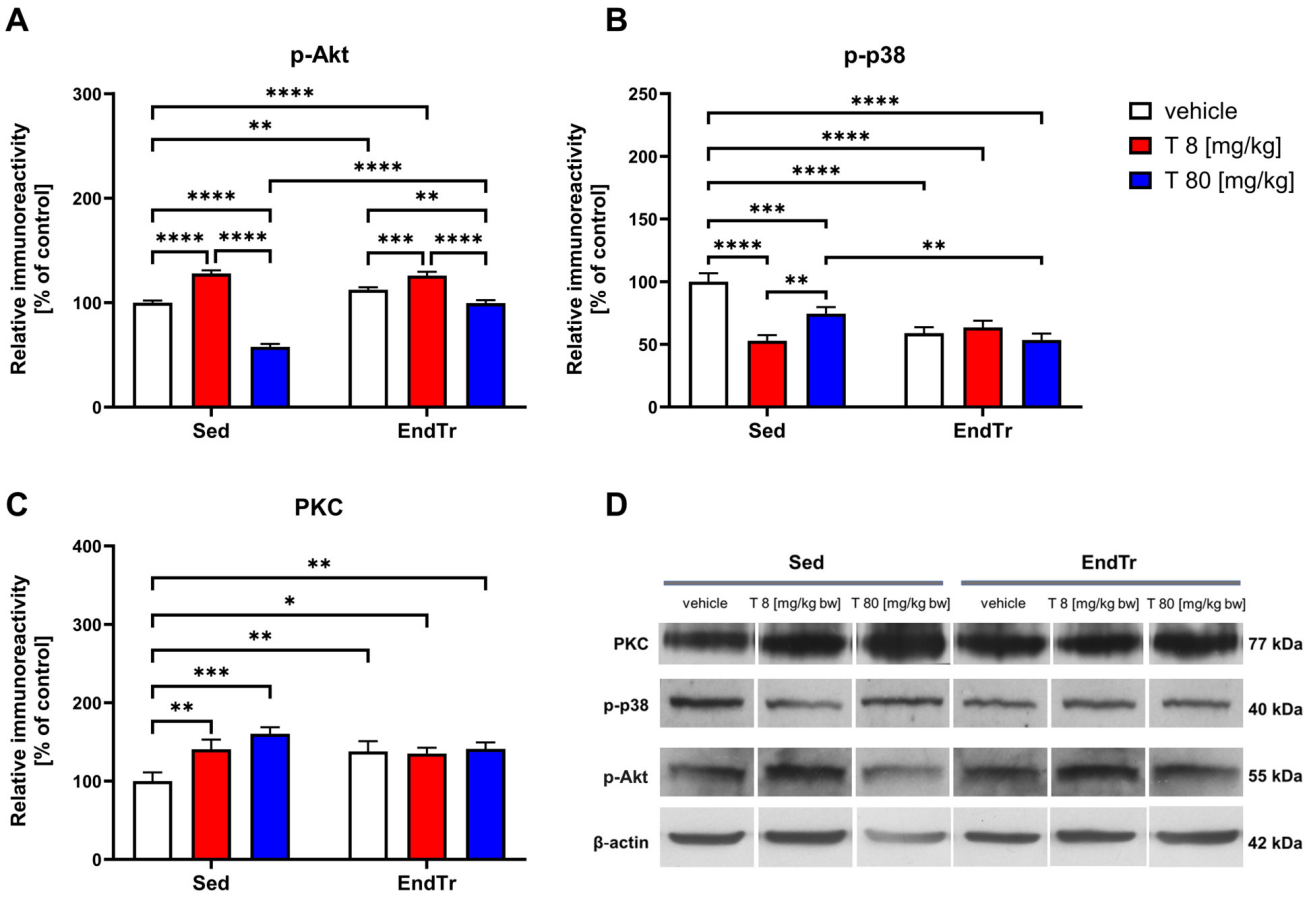
Six weeks of endurance training and testosterone treatment alter the spinal protein level of kinases in male adolescent rats. Western blotting analysis of protein relative immunoreactivity of p-Akt **(A)**, p-p38 **(B)**, and PKC **(C)** Testosterone treatment or endurance training, as well as simultaneous exposure to both interventions, lead to a decrease in p-p38 levels **(B)** On the contrary, the PKC levels increased **(C)** Furthermore, among sedentary rats, a dose-dependent changes in p-p38 level were demonstrated **(B)** Similar observations were noted in p-Akt levels, which were elevated following endurance training and/or low-dose testosterone (8 mg/kg) treatment. In both sedentary and trained rats, a high dose of testosterone (80 mg/kg) decreased the p-Akt levels compared to those treated with a lower dose (8 mg/kg) **(A)** Representative images of Western blotting analysis, cropped bands from different parts of the same blots are shown **(D)** Values are expressed as a percentage of the control. Data were analyzed with a two-way ANOVA and *post-hoc* Tukey's test: ****p < 0.0001, ***p < 0.001, **p < 0.01, *p < 0.05 (*n* = 19-20 for each experimental group).

The two-way ANOVA showed significant effects of endurance training (F[1,12] = 164.9, *p* < 0.0001) and testosterone treatment (F[2,12] = 430.5, *p* < 0.0001), and an interaction between these factors on p-Akt protein levels (F[2,12] = 91.59, *p* < 0.0001, Figure 3A, Table 2). The *post hoc* analysis showed a training-induced increase in the spinal level of p-Akt (Sed: veh vs. EndTr: veh: *p* < 0.0022). Training combined with testosterone in a low dose induced an even greater increase in the p-Akt level [Sed: veh vs. EndTr: T (8 mg/kg): *p* < 0.0001]. The level of p-Akt was significantly higher in both trained and untrained groups of animals treated with lower doses of testosterone in comparison to appropriate control groups treated with vehicle (Sed: veh vs. Sed: T [8 mg/kg]: *p* < 0.0001; EndTr: veh vs. EndTr: T [8 mg/kg]: *p* = 0.0009). Opposite, high-dose testosterone reduced the p-Akt levels (Sed: veh vs. Sed: T 80 mg/kg: *p* < 0.0001; EndTr: veh vs. EndTr T 80 mg/kg: *p* = 0.0015). Similarly to changes observed in the levels of most of the studied proteins, a dose-dependent effect of testosterone was observed. Namely, 80 mg/kg of testosterone decreased the spinal level of p-Akt in both trained and sedentary rats in comparison to the groups receiving lower doses of testosterone (Sed: T 8 vs. Sed: T [80 mg/kg]: *p* < 0.0001; EndTr: T 8 vs. EndTr: T 80 [mg/kg]: *p* < 0.0001). A significant reduction in the level of p-Akt was observed in the high dose of testosterone-treated sedentary rats compared to the training group [Sed: T 80 vs. EndTr: T 80 (mg/kg): *p* < 0.0001].

### 3.6 Both doses of testosterone and endurance training alone lowered the p-p38 protein level

The two-way ANOVA revealed significant effects of training and testosterone treatment on spinal p-p38 protein levels (F[1,12] = 46.35, *p* < 0.0001; F[2,12] = 25.34, *p* < 0.0001, respectively), and interaction between these factors (F[2,12] = 36.05, *p* < 0.0001, Figure 3B, Table 2). The *post hoc* analysis showed lowered p-p38 protein levels in the rat spinal cord of trained rats (Sed: veh vs. EndTr: veh: *p* < 0.0001) and sedentary rats treated with testosterone in both doses (Sed: veh vs. Sed: T 8 [mg/kg]: *p* < 0.0001, Sed: veh vs. Sed: T 80 [mg/kg]: *p* = 0.0008). Similar effects were observed when training was combined with testosterone treatment [Sed: veh vs. EndTr: T 8 and EndTr: T 80 (mg/kg): *p* < 0.0001]. Surprisingly, in rats receiving a low dose of testosterone, the level of p-p38 protein was significantly lower compared to the animals supplemented with a high dose [Sed: T 8 vs. Sed: T 80 (mg/kg): *p* = 0.0034]. The spinal level of p-p38 was decreased when comparing trained and non-trained animals receiving a high dose of testosterone [Sed: T 80 vs. EndTr: T 80 (mg/kg) = 0.0044].

### 3.7 Both doses of testosterone increased the PKC protein expression in the sedentary rats

Concerning the protein level of PKC, we observed significant effects of testosterone treatment (F[2,12] = 14.39, *p* = 0.0007) and testosterone x endurance training interaction (F[2,12] = 12.54, *p* = 0.0011), but with no effect of training (F[1,12] = 0.818, *p* = 0.383, Figure 3C). The *post hoc* analysis showed that training increased the spinal levels of PKC (Sed: veh vs. EndTr: veh: *p* = 0.0074). A higher level of PKC protein was noted in the sedentary animals treated with both doses of testosterone as compared to vehicle-treated (Sed: veh vs. Sed: T [8 mg/kg]: *p* = 0.0044, and Sed: veh vs. Sed: T [80 mg/kg] *p* = 0.0001). Similar effects were observed for the combination of training with testosterone supplementation in both doses as compared to vehicle-treated sedentary animals (Sed: veh vs. EndTr: T [8 mg/kg]: *p* = 0.0125, Sed: veh vs. EndTr: T [80 mg/kg] *p* = 0.004).

### 3.8 A high dose of testosterone, but no training lowered the spinal level of BDNF protein

**Figure 4:**
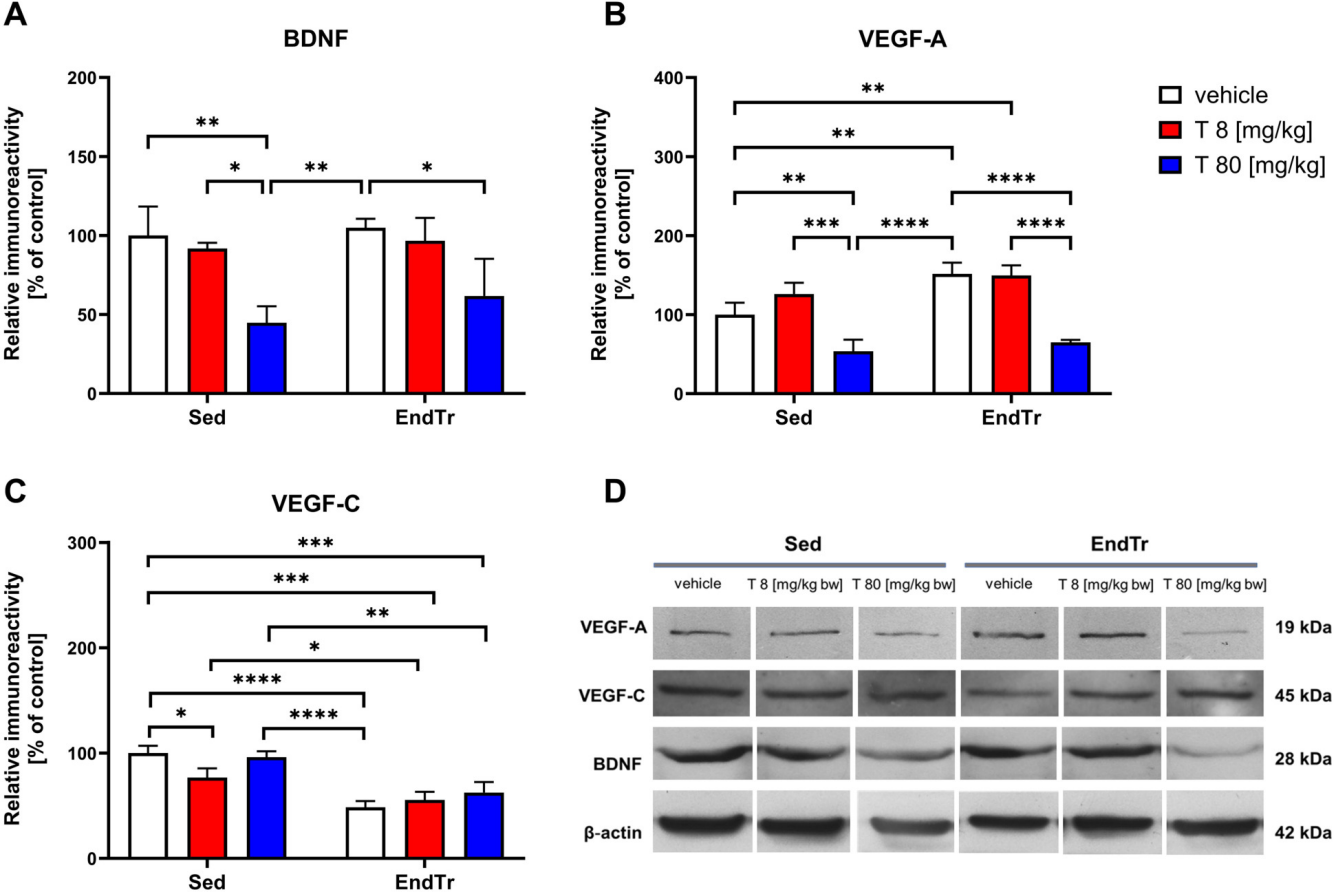
Six weeks of endurance training and testosterone treatment alter the spinal protein level of neurotrophins in male adolescent rats. Western blotting analysis of protein relative immunoreactivity of BDNF **(A)**, VEGF-A **(B)**, and VEGF-C **(C)** A high dose of testosterone (80 mg/kg) lowered the spinal level of BDNF **(A)**, and VEGF-A **(B)** proteins. Also, decreased level of VEGF-A were noted after high-dose testosterone treatment (80 mg/kg) combined with training **(B)** Furthermore, training reduced VEGF-C protein levels, regardless of vehicle and testosterone dose **(C)** Representative images of Western blotting analysis, cropped bands from different parts of the same blots are shown **(D)** Values are expressed as a percentage of the control. Data were analyzed with a two-way ANOVA and *post-hoc* Tukey's test: ****p < 0.0001, ***p < 0.001, **p < 0.01, *p < 0.05 (*n* = 19-20 for each experimental group).

The two-way ANOVA showed a significant effect of testosterone treatment on the spinal BDNF protein levels (F[2,12] = 19.09, *p* < 0.0002) with no significant impact of training (F[1,12] = 1.706, *p* = 0.216) and testosterone x training interaction (F[2,12]= 0.352, *p* = 0.709, Figure 4A, Table 2). The *post-hoc* analysis showed that a high dose of testosterone lowered the BDNF protein level in the untrained (sedentary) [Sed: veh vs. Sed: T (80 mg/kg): *p* = 0.0054] as well as in the trained rats [EndTr: veh vs. EndTr: T (80 mg/kg): *p* = 0.03]. Sedentary animals treated with 80 mg/kg of testosterone showed lower BDNF protein levels in comparison to sedentary receiving testosterone in the dose 8 mg/kg [Sed: T 8 vs. Sed: T 80 (mg/kg): *p* = 0.0172] as well as to vehicle-treated trained rats (Sed: T 80 mg/kg vs. EndTr:Veh: *p* = 0.0087).

### 3.9 A high dose of testosterone treatment alone and combined with training lowered the VEGF-A protein level

The two-way ANOVA showed significant effects of testosterone treatment and training on VEGF-A protein level in the spinal cord (F[2,12] = 63.09, *p* < 0.0001; F[1,12] = 21.78, *p* = 0.0005, respectively), and no significant interaction between these factors (F[2,12] = 3.803, *p* = 0.052, Figure 4B, Table 2). Endurance training increased spinal VEGF-A protein levels compared to sedentary rats (Sed: veh vs. EndTr: veh: *p* = 0.0041). Training combined with testosterone in a low dose [8 mg/kg] induced a significant increase of VEGF-A as compared to the sedentary group [Sed: veh vs. EndTr: T 8 (mg/kg): *p* = 0.0058]. A decrease in the level of VEGF-A protein was shown both in the groups of sedentary and training animals receiving a high dose of testosterone when comparing with appropriate control (Sed: veh vs. Sed: T 80 mg/kg: *p* = 0.0095; EndTr: veh vs. EndTr: T 80 mg/kg: *p* < 0.0001). A high-dose testosterone treatment decreased the spinal level of the VEGF-A in both trained and sedentary rats in comparison to the groups receiving a lower dose of testosterone (Sed: T 8 vs. Sed: T 80 mg/kg: *p* = 0.0002; EndTr: T 8 vs. EndTr: T 80 mg/kg: *p* < 0.0001). Further, similarly to changes observed in the levels of BDNF, the spinal level of VEGF-A in sedentary rats treated with a high dose of testosterone was decreased when compared to the vehicle-treated trained rats (Sed: T 80 mg/kg vs. EndTr: veh: *p* < 0.0001).

### 3.10 Training lowered the VEGF-C protein levels regardless of vehicle and testosterone in both doses

The two-way ANOVA revealed significant effects of testosterone treatment and training on the spinal level of VEGF-C protein (F[2,12] = 4.52, *p* = 0.0343; F[1,12] = 95.36, *p* < 0.0001, respectively), and interaction between these factors (F[2,12] = 5.87, *p* = 0.0166, Figure 4C, Table 2). A low dose of testosterone decreased VEGF-C protein level in the sedentary rats (Sed: veh vs. Sed: T 8 mg/kg: *p* = 0.0278). In all the trained rats, we observed lowered spinal levels of VEGF-C protein compared to the untrained rats receiving vehicle (Sed: veh vs. EndTr: veh: *p* < 0.0001, Sed: veh vs. EndTr: T 8 mg/kg: *p* = 0.0001, Sed: veh vs. EndTr: T 80 mg/kg: *p* = 0.0007) or testosterone in both doses (Sed: T 8 vs. EndTr: T 8 mg/kg: *p* = 0.0019, Sed: T 80 vs. EndTr: T 80 mg/kg: *p* = 0.0482). Regarding changes observed in the VEGF-A protein expression, VEGF-C level in sedentary rats treated with a high dose of testosterone was decreased when compared to the vehicle-treated trained rats (Sed: T 80 mg/kg vs. EndTr: veh: *p* < 0.0001).

Statistical details regarding western blot analysis are summarised in Table 2. Please find orginal blots in Supplementary File S1.

## 4 Discussion

Androgens, produced by the gonads or long-term administered, affect brain functions including motor and motivational behaviors, cognitive functions, and neurotransmitter release in dopamine midbrain circuits (25, 26). Supplementation with high doses of androgens is mostly used with strength training or bodybuilding. The effects of exercise training on brain function may also differ greatly depending on its maturity (27).

Thus, we used a well-known treadmill training scheme that was previously correlated with the improvement of endurance (28) as well as a variety of CNS effects, e.g., changes in the serotonergic system (29) or up-regulation of the NO/sGC/cGMP pathway in rat brain structures (30). Results from previous experimental studies applying a treadmill running in combination with testosterone treatment showed the potential risk to cardiac health (31) and significantly disturbed the liver antioxidant barrier and prooxidative-antioxidative balance in adolescent male rats (32).

Earlier, we conducted a study on the effect of 6 weeks of treadmill training and testosterone administration on blood-brain barrier (BBB) proteins in the spinal cords of young rats (23). We have suggested that an excessive supply of testosterone, but not endurance training alone, may impact both signal transduction in the endothelial cells of the CNS and the formation of tight junctions (TJs) in the blood-spinal cord barrier (BSCB) by decreasing the levels of several TJs proteins, including occludin, JAM-1, and VE-cadherin. The present study aimed to characterize the impact of combining testosterone treatment with endurance training on the protein expression of certain trophic factors and proteins related to the NO signaling pathway in the spinal cords of adolescent male rats. Our findings demonstrate that testosterone and endurance training modulate signaling pathways involved in neurovascular remodeling (see Figures 2–4). Major findings from the study are that (1) a high dose of testosterone increases the levels of proteins related to the NO signaling pathway (eNOS, nNOS, CGβ1, PKC), but decreases trophic factors (BDNF, VEGF) and p-Akt; (2) endurance training by itself increases the spinal protein levels of CGβ1, VEGF, and kinases—p-Akt and PKC, but decreases kinase p-p38 MAPK; and (3) combination of endurance training and high doses of testosterone enhances changes in the protein level of nNOS, p-p38 and p-Akt.

Although the positive effects of exercise training on the vasculature (and more specifically, endothelium-dependent vasodilation) have been demonstrated (33, 34), it is presently unclear what the ideal exercise type and dose are to produce favorable changes. Experimental studies showed that increasing the level of eNOS and NO production as a result of short-term endurance training (2–4 weeks) may be the initial stage in the adaptation of the vascular endothelium to physical exercise (35, 36) by producing a short-term buffer to the increased shear stress associated with exercise. After extended training, the increased production of NO and possibly other mediators induces structural changes in the vessels, increasing lumen diameter (37).

Our results suggest the increased activity of the protein NOS and an increase in eNOS protein expression at both doses (8 or 80 mg/kg body weight) of testosterone and nNOS protein after using a lower dose of testosterone. A combination of training with hormonal stimulation resulted in a similar effect. The level of nNOS is regulated by testosterone directly through androgen receptors (AR), or after being converted by the enzyme aromatase to estrogen via estrogen receptors (ERα and ERβ) (33). Endurance training alone and when combined with a lower dose of testosterone also caused an increase in the amount of the phosphorylated form of Akt kinase (p-Akt) and protein kinase C (PKC). A high dose of testosterone caused a decrease in the amount of p-Akt kinase compared to the control (sedentary) group. This dose-dependent divergence in Akt phosphorylation, clearly visible in Figure 3A, supports the notion that high testosterone might blunt beneficial training effects on intracellular signaling pathways. We acknowledge that the reduction in p-Akt levels following high-dose testosterone treatment is unexpected, given the known stimulatory role of testosterone on the IGF-1/PI3 K/Akt pathway (38). Testosterone is known to activate the Akt pathway via IGF-1/PI3 K signaling crosstalk in several tissues (39). Yin et al. (38) showed 3-week endurance training-induced muscle hypertrophy, which was mediated at least partly through IGF-1/IGF-1R-PI3 K/Akt-mTOR pathway.

The results presented in this paper partially correlate with the data obtained by Chalimoniuk (40). Increased NOS protein activity, increased protein expression of nNOS, and a higher CGβ1) level in the spinal cord after endurance training may indicate activation of the NO/GC/cGMP pathway. This effect also occurred when a combination of hormonal stimuli and training, as well as using only testosterone.

VEGF is a well-known factor considered to be important in the promotion of capillary growth in skeletal muscles exposed to increased activity (41, 42). Recently, Tang et al. (43) showed that treadmill running may provide a controlled cellular mechanism to increase VEGF expression in regions of the brain that are important for memory and sensory control. Increased VEGF expression in skeletal muscles and brain structures (hippocampus, cortex) may be important in coordinating the regulation of neurogenesis and angiogenesis in response to exercise and potentially may provide neuroprotection against ischemic or excitotoxic episodes. Among the main factors regulating VEGF protein expression are hypoxia, oxidative stress, growth factors, and cytokines, along with NO (44, 45). Increased muscle activity is known to upregulate nNOS in rat muscle (46) and eNOS in skeletal muscle arterioles (47). In the study by Suzuki et al. (2005), the authors noted that the simultaneous use of five weeks of running training and L-arginine supplementation in male rats resulted in an increased capillarization in the soleus muscle and subendocardial layer of the myocardium. Expression of VEGF protein and endothelial nitric oxide synthase (eNOS) in soleus muscle and myocardium increased significantly when used concurrently with L-arginine supplementation and endurance training (48). Here, we showed that combining endurance training with testosterone in a low dose resulted in similar effects induced by 8 mg/kg testosterone itself. Namely, we noted decreased protein levels of VEGF-A, VEGF-C, and p-p38 and increased protein levels of p-Akt, PKC, nNOS, and CGβ1. However, our data (Figure 4) also demonstrate that a high dose of testosterone reverses these effects, indicating possible adverse consequences on neurovascular health. This observation, though preliminary, calls for further investigation into dose-dependent risk profiles.

The exercise-induced upregulation of hippocampal BDNF level was confirmed in many animal studies (49–52). Physical exercise-induced increases in BDNF expression were also noted in the frontal cortex (53) and the spinal cord (52). For example, in the study by Perreau et al. (2005), the authors demonstrated increased BDNF mRNA and protein levels in the thoracic and lumbar sections of the spinal cord of rats subjected to wheel running for 21 days (54). Forced training schemes might induce stress reactions in animals. According to Huang et al., who showed the importance of 1-week pre-familiarization, we also adapted rats before the treadmill training. However, the lack of assessment of stress markers (for example, serum corticosterone levels) is a limitation of the present study. Contrary to the above studies, we did not observe the effect of training on the spinal BDNF protein level. Some aspects of the training protocol or animal model might explain this discrepancy. Endurance training, particularly when prolonged, can increase BDNF release from the brain, potentially influencing spinal cord function (55). However, it should be acknowledged that the increases in BDNF protein in the spinal cord were observed mainly in models of spinal cord injuries (56, 57). Further, endurance training specificity is critically important since it affects the subsequent adaptations of the tissue. The adaptations created by resistance and endurance training have conflicting outcomes (58). Androgen-dependent regulation of BDNF protein was well described in the literature using castrated rats (59–61). Castrated adult rats showed retracted dendrites of androgen-sensitive motoneurons of the spinal nucleus of the bulbocavernosus (SNB). BDNF, via activation of tyrosine receptor kinase B (trkB), has been implicated in mediating androgen effects on SNB dendrites (59). A high dose of testosterone, by reducing the spinal BDNF protein level, may have an adverse effect on the functioning of the CNS. Our findings (Figure 4A) revealed a significant reduction in spinal BDNF protein levels following high-dose testosterone, regardless of training. This suggests a suppressive effect that may override training-induced neuroplastic benefit.

### 4.1 Limitations of the study

It should be acknowledged that some of the mechanistic interpretations of kinase regulation in the present study remain incomplete. The inability to distinguish changes in kinase phosphorylation (activation state) from total protein immunoreactivity due to the lack of total p38 and Akt quantification may be interpreted as one of the limitations. Since CREB is a transcription factor of neuroplasticity-related genes, including BDNF and VEGF, its phosphorylation (p-CREB) is downstream of multiple signaling pathways investigated in this study (e.g., NO/cGMP/PKG, Akt, p38 MAPK). However, the absence of CREB and p-CREB data also limits mechanistic insight into the transcriptional regulation of BDNF and VEGF in response to testosterone and endurance training, limiting our ability to mechanistically link upstream signaling changes (NO, Akt, p38) to the regulation of BDNF and VEGF expression. Measurements of both total and phosphorylated kinase levels should be warranted in future studies. This approach will support differentiation between transcriptional and post-translational effects, providing a more nuanced understanding of cellular signaling pathways.

## 5 Conlusions

In summary, our findings reinforce the view that testosterone and endurance training modulate overlapping but not necessarily synergistic molecular pathways in the spinal cord. The absence of statistically significant interactions in several markers underscores this independent modulation**.** At least some of the effects of endurance training and testosterone may be related to the intensity of NO-related signal transmission and protein kinase systems. Endurance training has a positive effect on the VEGF-A protein expression in the spinal cord; however, it is not able to eliminate the unfavorable effects of high doses of testosterone.

## Data Availability

The original contributions presented in the study are included in the article/Supplementary Material, further inquiries can be directed to the corresponding author.

## References

[1] Brooks GA. Bioenergetics of exercising humans. Compr Physiol. (2012) 2(1):537–62. 10.1002/cphy.c110007 23728979

[2] Hughes DC Ellefsen S Baar K. Adaptations to endurance and strength training. Cold Spring Harb Perspect Med. (2018) 8(6):a029769. Published 2018 Jun 1. 10.1101/cshperspect.a029769 28490537 PMC5983157

[3] Rønnestad BR Mujika I. Optimizing strength training for running and cycling endurance performance: a review. Scand J Med Sci Sports. (2014) 24(4):603–12. 10.1111/sms.12104 23914932

[4] Graham MR Davies B Grace FM Kicman A Baker JS. Anabolic steroid use: patterns of use and detection of doping. Sports Med. (2008) 38(6):505–25. 10.2165/00007256-200838060-00005 18489196

[5] Bahrke MS Yesalis CE Brower KJ. Anabolic-androgenic steroid abuse and performance-enhancing drugs among adolescents. Child Adolesc Psychiatr Clin N Am. (1998) 7(4):821–38. 10.1016/S1056-4993(18)30214-1 9894044

[6] Kokkevi A Fotiou A. Chilevascience.2005.03.026.

[7] Gestsdottir S Kristjansdottir H Sigurdsson H Sigfusdottir ID. Prevalence, mental health and substance use of anabolic steroid users: a population-based study on young individuals. Scand J Public Health. (2021) 49(5):555–62. 10.1177/1403494820973096 33280527

[8] Musienko PE Zelenin PV Lyalka VF Gerasimenko YP Orlovsky GN Deliagina TG. Spinal and supraspinal control of the direction of stepping during locomotion. J Neurosci. (2012) 32(48):17442–53. 10.1523/JNEUROSCI.3757-12.2012 23197735 PMC3521535

[9] Leiras R Cregg JM Kiehn O. Brainstem circuits for locomotion. Annu Rev Neurosci. (2022) 45:63–85. 10.1146/annurev-neuro-082321-025137 34985919

[10] MacKay-Lyons M. Central pattern generation of locomotion: a review of the evidence. Phys Ther. (2002) 82(1):69–83. 10.1093/ptj/82.1.69 11784280

[11] Takakusaki K. Neurophysiology of gait: from the spinal cord to the frontal lobe. Mov Disord. (2013) 28(11):1483–91. 10.1002/mds.25669 24132836

[12] Kyriakatos A Molinari M Mahmood R Grillner S Sillar KT El Manira A. Nitric oxide potentiation of locomotor activity in the spinal cord of the lamprey. J Neurosci. (2009) 29(42):13283–91. 10.1523/JNEUROSCI.3069-09.2009 19846716 PMC6665181

[13] Cossenza M Socodato R Portugal CC Bednarski T Maczewski M Langfort J Nitric oxide in the nervous system: biochemical, developmental, and neurobiological aspects. Vitam Horm. (2014) 96:79–125. 10.1016/B978-0-12-800254-4.00005-2 25189385

[14] Shimada Y Kaji K Ito H. Heterogeneous responses of human endothelial cells to tumor necrosis factor with respect to growth inhibition. Artery. (1991) 18(5):268–84. 1929886

[15] Villanueva C Giulivi C. Subcellular and cellular locations of nitric oxide synthase isoforms as determinants of health and disease. Free Radic Biol Med. (2010) 49(3):307–16. 10.1016/j.freeradbiomed.2010.04.004 20388537 PMC2900489

[16] Boehning D Snyder SH. Novel neural modulators. Annu Rev Neurosci. (2003) 26:105–31. 10.1146/annurev.neuro.26.041002.131047 14527267

[17] Endres M Gertz K Lindauer U Katchanov J Schultze J Schröck H Mechanisms of stroke protection by physical activity. Ann Neurol. (2003) 54(5):582–90. 10.1002/ana.10722 14595647

[18] Pietrelli A López-Costa JJ Goñi R López EM Brusco A Basso N. Effects of moderate and chronic exercise on the nitrergic system and behavioral parameters in rats. Brain Res. (2011) 1389:71–82. 10.1016/j.brainres.2011.03.005 21396922

[19] Chen MJ Ivy AS Russo-Neustadt AA. Nitric oxide synthesis is required for exercise-induced increases in hippocampal BDNF and phosphatidylinositol 3’ kinase expression. Brain Res Bull. (2006) 68(4):257–68. 10.1016/j.brainresbull.2005.08.013 16377431

[20] Xiong H Yamada K Han D Nabeshima T Enikolopov G Carnahan J Mutual regulation between the intercellular messengers nitric oxide and brain-derived neurotrophic factor in rodent neocortical neurons. Eur J Neurosci. (1999) 11(5):1567–76. 10.1046/j.1460-9568.1999.00567.x 10215909

[21] Berchtold NC Chinn G Chou M Kesslak JP Cotman CW. Exercise primes a molecular memory for brain-derived neurotrophic factor protein induction in the rat hippocampus. Neuroscience. (2005) 133(3):853–61. 10.1016/j.neuroscience.2005.03.026 15896913

[22] Uysal N Kiray M Sisman AR Camsari UM Gencoglu C Baykara B Effects of voluntary and involuntary exercise on cognitive functions, and VEGF and BDNF levels in adolescent rats. Biotech Histochem. (2015) 90(1):55–68. 10.3109/10520295.2014.946968 25203492

[23] Nierwińska K Nowacka-Chmielewska M Bernacki J Jagsz S Chalimoniuk M Langfort J The effect of endurance training and testosterone supplementation on the expression of blood spinal cord barrier proteins in rats. PLoS One. (2019) 14(2):e0211818. 10.1371/journal.pone.0211818 30742658 PMC6370194

[24] Sadowska-Krępa E Kłapcińska B Jagsz S High-dose testosterone enanthate supplementation boosts oxidative stress, but exerts little effect on the antioxidant barrier in sedentary adolescent male rat liver. Pharmacol Rep. (2017) 69(4):673–8. 10.1016/j.pharep.2017.02.023 28531762

[25] de Souza Silva MA Mattern C Topic B Buddenberg TE Huston JP. Dopaminergic and serotonergic activity in neostriatum and nucleus accumbens enhanced by intranasal administration of testosterone. Eur Neuropsychopharmacol. (2009) 19(1):53–63. 10.1016/j.euroneuro.2008.08.003 18818056

[26] Thiblin I Finn A Ross SB Stenfors C. Increased dopaminergic and 5-hydroxytryptaminergic activities in male rat brain following long-term treatment with anabolic androgenic steroids. Br J Pharmacol. (1999) 126(6):1301–6. 10.1038/sj.bjp.0702412 10217522 PMC1565900

[27] Zhang M Jia J Yang Y Zhang L Wang X. Effects of exercise interventions on cognitive functions in healthy populations: a systematic review and meta-analysis. Ageing Res Rev. (2023) 92:102116. 10.1016/j.arr.2023.102116 37924980

[28] Dobrzyn P Pyrkowska A Duda MK Bednarski T Maczewski M Langfort J Expression of lipogenic genes is upregulated in the heart with exercise training-induced but not pressure overload-induced left ventricular hypertrophy. Am J Physiol Endocrinol Metab. (2013) 304(12):E1348–58. 10.1152/ajpendo.00603.2012 23632628

[29] Langfort J Barańczuk E Pawlak D Chalimonik M Lukacova N Marsala J The effect of endurance training on regional serotonin metabolism in the brain during early stage of detraining period in the female rat. Cell Mol Neurobiol. (2006) 26(7-8):1327–42. 10.1007/s10571-006-9065-5 16897368 PMC11520764

[30] Chalimoniuk M Chrapusta SJ Lukačova N Langfort J. Endurance training upregulates the nitric oxide/soluble guanylyl cyclase/cyclic guanosine 3’,5'-monophosphate pathway in the striatum, midbrain and cerebellum of male rats. Brain Res. (2015) 1618:29–40. 10.1016/j.brainres.2015.05.020 26006108

[31] Sadowska-Krępa E Kłapcińska B Jagsz S Sobczak A Chrapusta SJ Chalimoniuk M High-dose testosterone propionate treatment reverses the effects of endurance training on myocardial antioxidant defenses in adolescent male rats. Cardiovasc Toxicol. (2011) 11(2):118–27. 10.1007/s12012-011-9105-3 21312070 PMC3085793

[32] Sadowska-Krępa E Kłapcińska B Nowara A High-dose testosterone supplementation disturbs liver pro-oxidant/antioxidant balance and function in adolescent male Wistar rats undergoing moderate-intensity endurance training. PeerJ. (2020) 8:e10228. Published 2020 November 19. 10.7717/peerj.10228 33240609 PMC7680624

[33] Green DJ Maiorana A O'Driscoll G Taylor R. Effect of exercise training on endothelium-derived nitric oxide function in humans. J Physiol. (2004) 561(Pt 1):1–25. 10.1113/jphysiol.2004.068197 15375191 PMC1665322

[34] Goto C Higashi Y Kimura M Noma K Hara K Nakagawa K Effect of different intensities of exercise on endothelium-dependent vasodilation in humans: role of endothelium-dependent nitric oxide and oxidative stress. Circulation. (2003) 108(5):530–5. 10.1161/01.CIR.0000080893.55729.28 12874192

[35] Sun D Huang A Koller A Kaley G. Short-term daily exercise activity enhances endothelial NO synthesis in skeletal muscle arterioles of rats. J Appl Physiol (1985). (1994) 76(5):2241–7. 10.1152/jappl.1994.76.5.2241 7520432

[36] Sun D Huang A Koller A Kaley G. Adaptation of flow-induced dilation of arterioles to daily exercise. Microvasc Res. (1998) 56(1):54–61. 10.1006/mvre.1998.2083 9683563

[37] Prior BM Lloyd PG Yang HT Terjung RL. Exercise-induced vascular remodeling. Exerc Sport Sci Rev. (2003) 31(1):26–33. 10.1097/00003677-200301000-00006 12562167

[38] Yin L Lu L Lin X Wang X. Crucial role of androgen receptor in resistance and endurance trainings-induced muscle hypertrophy through IGF-1/IGF-1R- PI3 K/Akt- mTOR pathway. Nutr Metab (Lond). (2020) 17:26. Published 2020 March 30. 10.1186/s12986-020-00446-y 32256674 PMC7106900

[39] White JP Gao S Puppa MJ Sato S Welle SL Carson JA. Testosterone regulation of akt/mTORC1/FoxO3a signaling in skeletal muscle. Mol Cell Endocrinol. (2013) 365(2):174–86. 10.1016/j.mce.2012.10.019 23116773 PMC3529800

[40] Duckles SP Miller VM. Hormonal modulation of endothelial NO production. Pflugers Arch. (2010) 459(6):841–51. 10.1007/s00424-010-0797-1 20213497 PMC2865573

[41] Chalimoniuk M Głowacka J Zabielna A Eckert A Strosznajder JB. Nitric oxide alters arachidonic acid turnover in brain cortex synaptoneurosomes. Neurochem Int. (2006) 48(1):1–8. 10.1016/j.neuint.2005.08.011 16216387

[42] Gavin TP Spector DA Wagner H Breen EC Wagner PD. Nitric oxide synthase inhibition attenuates the skeletal muscle VEGF mRNA response to exercise. J Appl Physiol (1985). (2000) 88(4):1192–8. 10.1152/jappl.2000.88.4.1192 10749807

[43] Hoier B Hellsten Y. Exercise-induced capillary growth in human skeletal muscle and the dynamics of VEGF. Microcirculation. (2014) 21(4):301–14. 10.1111/micc.12117 24450403

[44] Tang K Xia FC Wagner PD Breen EC. Exercise-induced VEGF transcriptional activation in brain, lung and skeletal muscle. Respir Physiol Neurobiol. (2010) 170(1):16–22. 10.1016/j.resp.2009.10.007 19853064 PMC2826189

[45] Ferrara N Davis-Smyth T. The biology of vascular endothelial growth factor. Endocr Rev. (1997) 18(1):4–25. 10.1210/edrv.18.1.0287 9034784

[46] Dyakova EY Kapilevich LV Shylko VG Popov SV Anfinogenova Y. Physical exercise associated with NO production: signaling pathways and significance in health and disease. Front Cell Dev Biol. (2015) 3:19. Published 2015 April 2. 10.3389/fcell.2015.00019 25883934 PMC4382985

[47] Song W Kwak HB Kim JH Lawler JM. Exercise training modulates the nitric oxide synthase profile in skeletal muscle from old rats. J Gerontol A Biol Sci Med Sci. (2009) 64(5):540–9. 10.1093/gerona/glp021 19304939 PMC2800810

[48] Sun D Huang A Koller A Kaley G. Enhanced NO-mediated dilations in skeletal muscle arterioles of chronically exercised rats. Microvasc Res. (2002) 64(3):491–6. 10.1006/mvre.2002.2450 12453444

[49] Suzuki J. Microvascular angioadaptation after endurance training with L-arginine supplementation in rat heart and hindleg muscles. Exp Physiol. (2005) 90(5):763–71. 10.1113/expphysiol.2005.031138 16002497

[50] Wrann CD White JP Salogiannnis J Laznik-Bogoslavski D Wu J Ma D Exercise induces hippocampal BDNF through a PGC-1*α*/FNDC5 pathway. Cell Metab. (2013) 18(5):649–59. 10.1016/j.cmet.2013.09.008 24120943 PMC3980968

[51] Zhou W Barkow JC Freed CR. Running wheel exercise reduces *α*-synuclein aggregation and improves motor and cognitive function in a transgenic mouse model of Parkinson’s disease. PLoS One. (2017) 12(12):e0190160. Published 2017 December 22. 10.1371/journal.pone.0190160 29272304 PMC5741244

[52] Naghibi S Shariatzadeh Joneydi M Barzegari A Davoodabadi A Ebrahimi A Eghdami E Treadmill exercise sex-dependently alters susceptibility to depression-like behaviour, cytokines and BDNF in the hippocampus and prefrontal cortex of rats with sporadic Alzheimer-like disease. Physiol Behav. (2021) 241:113595. 10.1016/j.physbeh.2021.113595 34536437

[53] Fang ZH Lee CH Seo MK Cho H Lee JG Lee BJ Effect of treadmill exercise on the BDNF-mediated pathway in the hippocampus of stressed rats. Neurosci Res. (2013) 76(4):187–94. 10.1016/j.neures.2013.04.005 23665137

[54] Perreau VM Adlard PA Anderson AJ Cotman CW. Exercise-induced gene expression changes in the rat spinal cord. Gene Expr. (2005) 12(2):107–21. 10.3727/000000005783992115 15892452 PMC6009109

[55] Gómez-Pinilla F Ying Z Opazo P Roy RR Edgerton VR. Differential regulation by exercise of BDNF and NT-3 in rat spinal cord and skeletal muscle. Eur J Neurosci. (2001) 13(6):1078–84. 10.1046/j.0953-816x.2001.01484.x 11285004

[56] Hayashi N Himi N Nakamura-Maruyama E Okabe N Sakamoto I Hasegawa T Improvement of motor function induced by skeletal muscle contraction in spinal cord-injured rats. Spine J. (2019) 19(6):1094–105. 10.1016/j.spinee.2018.12.012 30583107

[57] Jung SY Seo TB Kim DY. Treadmill exercise facilitates recovery of locomotor function through axonal regeneration following spinal cord injury in rats. J Exerc Rehabil. (2016) 12(4):284–92. Published 2016 August 31. 10.12965/jer.1632698.349 27656624 PMC5031384

[58] Tanaka H Swensen T. Impact of resistance training on endurance performance. A new form of cross-training? Sports Med. (1998) 25(3):191–200. 10.2165/00007256-199825030-00005 9554029

[59] Ottem EN Beck LA Jordan CL Breedlove SM. Androgen-dependent regulation of brain-derived neurotrophic factor and tyrosine kinase B in the sexually dimorphic spinal nucleus of the bulbocavernosus. Endocrinology. (2007) 148(8):3655–65. 10.1210/en.2007-0308 17463054

[60] Zhang KJ Ramdev RA Tuta NJ Spritzer MD. Dose-dependent effects of testosterone on spatial learning strategies and brain-derived neurotrophic factor in male rats. Psychoneuroendocrinology. (2020) 121:104850. 10.1016/j.psyneuen.2020.104850 32892065 PMC7572628

[61] Fanaei H Karimian SM Sadeghipour HR Hassanzade G Kasaeian A Attari F Testosterone enhances functional recovery after stroke through promotion of antioxidant defenses, BDNF levels and neurogenesis in male rats. Brain Res. (2014) 1558:74–83. 10.1016/j.brainres.2014.02.028 24565925

