# Supplementary material for: Dose-dependent effects of testosterone on proteins related to nitric oxide signaling pathway and trophic factors in the spinal cord of adolescent trained rats
Source: Front Sports Act Living. 2025 Jul 22;7:1635517. doi: 10.3389/fspor.2025.1635517 (PMC12322838; doi:10.3389/fspor.2025.1635517)
Supplement: Supplementary file 1 [file Table1.docx]

**Dose-dependent effects of testosterone on proteins related to nitric oxide signaling pathway and trophic factors in the spinal cord of adolescent trained rats.**

**Katarzyna Nierwińska^1^, Konstancja Grabowska^1^, Małgorzata Chalimoniuk^2^, Sławomir Jagsz^3^, Józef Langfort^4^,  Andrzej Małecki^1^, Marta Nowacka-Chmielewska^1^**

1 Laboratory of Molecular Biology, Institute of Physiotherapy and Health Sciences, Academy of Physical Education in Katowice, Mikolowska 72a, 40-065 Katowice, Poland

2 Faculty in Biala Podlaska, Jozef Pilsudski University of Physical Education in Warsaw, 21-500 Warszawa, Poland.

3 Department of Biochemistry, The Jerzy Kukuczka Academy of Physical Education, Katowice, Poland.

4 Department of Sports Nutrition, The Jerzy Kukuczka Academy of Physical Education in Katowice, Mikolowska 72a, 40-065 Katowice, Poland. j.langfort@awf.katowice.pl.

Correspondence: Katarzyna Nierwińska; e-mail: k.nierwinska@awf.katowice.pl


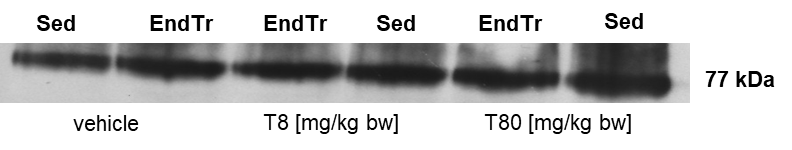


**Fig. 1** Visualization of PKC bands.


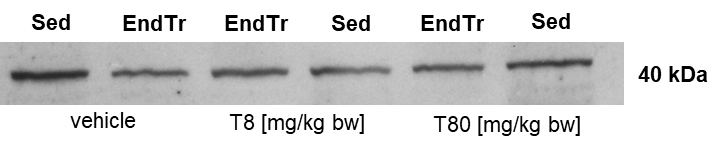


**Fig. 2** Visualization of p-p38 bands.


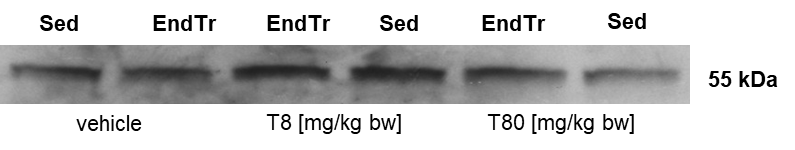


**Fig. 3** Visualization of pAKT bands.


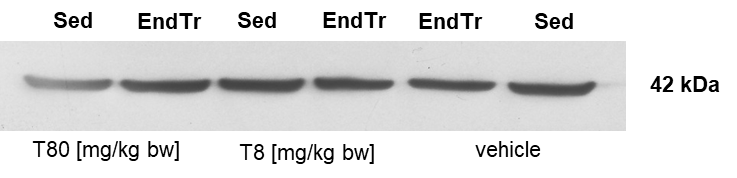


**Fig. 4** Visualization of β-actin bands as a loading control to PKC, p-p38, and pAKT.

**
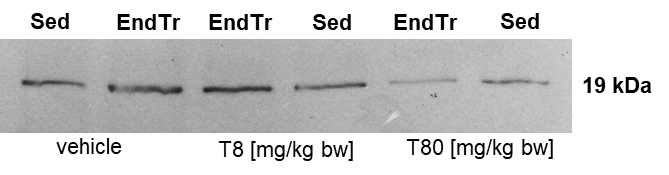
**

**Fig. 5** Visualization of VEGF-A bands.


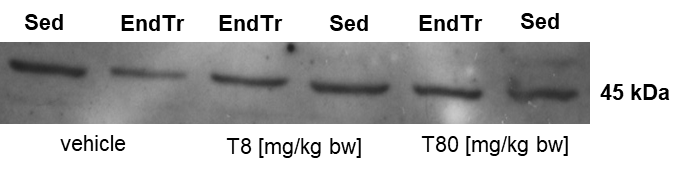


**Fig. 6** Visualization of VEGF-C bands.


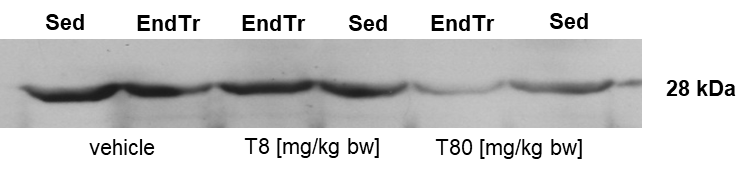


**Fig. 7** Visualization of BDNF bands.


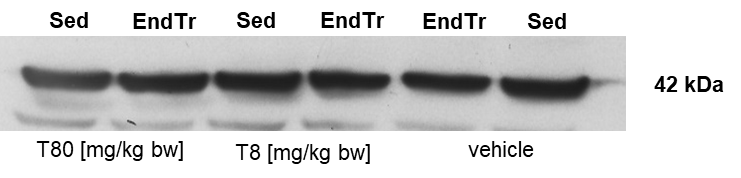


**Fig. 8** Visualization of β-actin bands as a loading control to VEGF-A, VEGF-C, and BDNF.

**
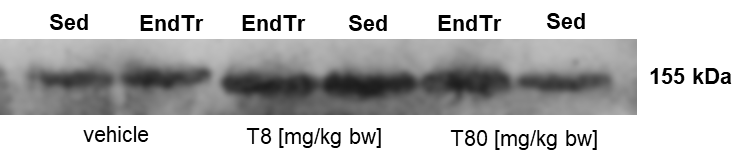
**

**Fig. 9** Visualization of nNOS bands.


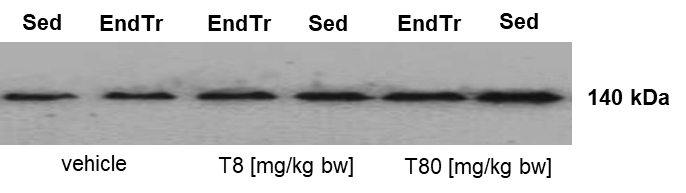


**Fig. 10** Visualization of eNOS bands


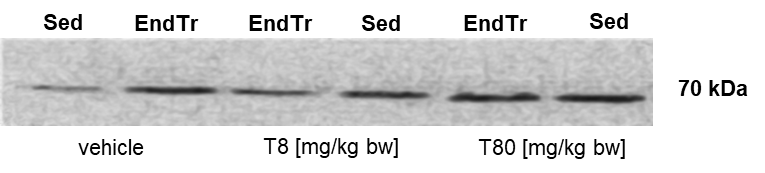


**Fig. 11** Visualization of CGβ1 bands.


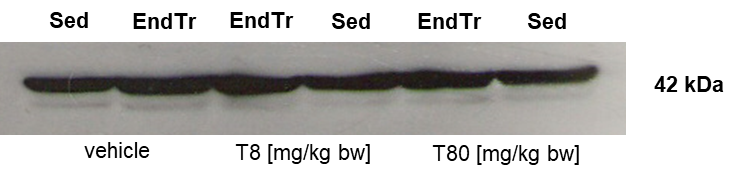


**Fig. 12** Visualization of β-actin bands as a loading control to nNOS, eNOS, and CGβ1.
